# Applying Supervised Machine Learning to Effusion Analysis for the Diagnosis of Feline Infectious Peritonitis

**DOI:** 10.3390/bioengineering13020127

**Published:** 2026-01-23

**Authors:** Dawn E. Dunbar, Simon A. Babayan, Sarah Krumrie, Sharmila Rennie, Elspeth M. Waugh, Margaret J. Hosie, William Weir

**Affiliations:** 1 School of Biodiversity, One Health and Veterinary Medicine, College of Medical, Veterinary and Life Sciences, University of Glasgow, Glasgow G12 8QQ, UK; simon.babayan@glasgow.ac.uk (S.A.B.); sharmila.rennie@glasgow.ac.uk (S.R.); elspeth.waugh@glasgow.ac.uk (E.M.W.); willie.weir@glasgow.ac.uk (W.W.); 2 Dunbia, The Summit, Pynes Hill, Exeter EX2 5WS, UK; 3 MRC-University of Glasgow Centre for Virus Research, College of Medical, Veterinary and Life Sciences, University of Glasgow, Glasgow G12 8QQ, UK; margaret.hosie@glasgow.ac.uk

**Keywords:** feline infectious peritonitis, feline coronavirus, machine learning, diagnostic, modelling, predictive

## Abstract

Feline infectious peritonitis (FIP) is a major disease of cats which, unless promptly diagnosed and treated, is invariably fatal. Although it has long been recognised that the condition is the result of an aberrant immune response to infection with feline coronavirus, there remain significant gaps in our understanding of its pathogenesis. Consequently, diagnosis is complex and relies on the combined interpretation of numerous clinical signs and laboratory biomarkers, many of which are non-specific. In the case of effusive FIP, a commonly encountered acute form of the disease where body cavity effusions develop; the interpretation of fluid analysis results is key to diagnosing the condition. We hypothesised that machine learning could be applied to fluid analysis test data in order to help diagnose effusive FIP. Thus, historical test records from a veterinary laboratory dataset of 718 suspected cases of effusive disease were identified, representing 336 cases of FIP and 382 cases that were determined not to be FIP. This dataset was used to train an ensemble model to predict disease status based on clinical observations and laboratory features. Our model predicts the correct disease state with an accuracy of 96.51%, an area under the receiver operator curve of 96.48%, a sensitivity of 98.85% and a specificity of 94.12%. This study demonstrates that machine learning can be successfully applied to the interpretation of fluid analysis results to accurately detect cases of effusive FIP. Thus, this method has the potential to be utilised in a veterinary diagnostic laboratory setting to standardise and improve service provision.

## 1. Introduction

Feline infectious peritonitis is one of the most difficult to diagnose viral diseases of cats, and it was, until recently, one of the most common infectious causes of mortality in felids [1,2]. Feline coronavirus (FCoV) is the infectious agent responsible for infection and subsequent disease. This virus is ubiquitous in the feline population and can infect both domestic and wild felids, causing FIP [3], although typically the virus only causes a sub-clinical or mild-moderate enteritis [4,5,6]. A number of virally encoded genes have been implicated in determining viral cell tropism. In enteric infection the virus targets enterocytes whereas FIP is believed to develop following a switch in viral tropism, resulting in the virus replicating to high titres in the monocyte/macrophage lineages of leukocytes [1,7] as a result of de novo mutation inside the host. The factors influencing the tropism switch and subsequent changes in virulence have yet to be fully elucidated [8,9,10,11,12]. Although FCoV infection occurs globally with a high prevalence (ranging from 25% to 100%) depending on environmental and husbandry factors, FIP only occurs in between 5% and 10% of infected individuals [13,14,15]. Whilst uncommon, epizootic outbreaks and appearance of novel recombinant viral strains with epizootic potential have been described recently. [16,17] The disease typically presents as one of three clinical manifestations, namely ‘effusive’, ‘non-effusive’ or a ‘mixed’ presentation. Effusive FIP is associated with the presence of a cavity effusion [18], a clinical sign which is not evident in non-effusive cases. A mixed presentation is generally described where clinical signs are consistent with non-effusive disease and then progress, with the development of an effusion. Effusions are the abnormal accumulation of fluid in body cavities, typically abdominal, pleural/thoracic or pericardial spaces [19,20,21,22,23,24]. In addition to causing visible abdominal distension, effusions may induce clinical signs such as dyspnoea, together contributing to lethargy and discomfort on movement, which may be more generally associated with the disease. FIP is likely to be one of the top differential diagnoses when a cat is presented to a clinical practitioner with the presence of an effusion as a major clinical sign [25]. Effusive FIP is considered to be the acute form of the disease, with rapid development of clinical signs, while non-effusive disease generally has a more insidious development, often over weeks to months.

For decades, presumptive diagnosis has been based on the assimilation of signalment, clinical signs, serology, haematology and biochemistry data [18,25]. However, in practice, determining a diagnosis of FIP is challenging because the laboratory data primarily evidence a generalised inflammatory response which may be common to other infectious disease, such as toxoplasmosis. Although advances in molecular methods since the turn of this century have allowed for the development of diagnostic tools which supplement traditional testing, there is still no conclusive stand-alone test for FIP diagnosis [26,27]. There have been improvements in the diagnosis of non-effusive FIP, arguably the more difficult form of the disease to diagnose, following the description of several real-time reverse-transcriptase quantitative polymerase chain reaction (RT-qPCR) assays that permit the identification of FCoV within tissues [28,29,30,31]. Similar assays have been developed for detecting the virus in peritoneal, pleural and pericardial effusions for the diagnosis of effusive disease [32,33,34,35,36]. While RT-qPCR is of great diagnostic utility in detecting cases of FIP, it does have limitations and should not be used as a standalone test for the disease. For example, when testing for FCoV in mesenteric lymph nodes [28] and cavity effusions, there exists an appreciable risk of blood contamination during sample collection, and the detection of FCoV in blood is not considered to be reliable for the diagnosis of FIP. [37,38] In addition, inter-laboratory differences in nucleic acid extraction methods will inevitably influence test sensitivity. Effusion samples may also be tested using immunocytochemistry (ICC) to directly detect the pathogen in cells [39,40,41,42,43,44]; however, this method is not commonly available within the United Kingdom (UK). Despite the availability of these tests, immunohistochemistry (IHC) is considered the only routinely available, definitive method of ante-mortem diagnosis of FIP for whichever manifestation of disease [29,45,46,47].

Developing a presumptive diagnosis of effusive FIP involves the application of similar tests to those utilised for non-effusive disease [25,48,49,50]. Whilst haematology and blood biochemistry may be performed as part of a general clinical investigation, in cases of suspected effusive disease, analysis of the effusion is often prioritised, as it is a relatively low-cost approach and may yield considerable diagnostic insight [25,51]. A typical testing profile for effusive FIP may include measurement of FCoV antibody titre, alpha-1-glycoprotein, total protein, albumin, globulins, albumin/globulin ratio and cytological analysis of the effusion, including differential cell counts. If the balance of evidence of these test results combined with the signalment and clinical history provide a strong suspicion of disease, the addition of RT-qPCR of the effusion and/or ICC may be indicated to strengthen support for a diagnosis [25]. Conversely, analysis of the effusion and additional follow-up testing may provide sufficient evidence to exclude FIP from the list of differential diagnoses, and in some cases may provide evidence to support an alternative explanation for the clinical signs.

Machine learning (ML) is a branch of artificial intelligence that has, in recent decades, been increasingly employed within medicine, and more recently in veterinary medicine, to explore vast clinicopathological datasets [52,53,54,55,56]. These can be analysed to provide new insights from the interpretation of laboratory markers, thereby informing and improving novel diagnostic and prognostic algorithms. Pfannschmidt et al. used FIP as an example to illustrate how game theory, a form of semi-supervised machine learning, could be applied to a clinical dataset to discriminate disease, although they used only a limited number of diagnostic tests to evaluate prediction of disease [57]. Subsequent studies seeking to apply machine learning to diagnosis of FIP have also evaluated limited data, one assessing a panel of clinical signs in isolation [58], and another assessing RT-qPCR and IHC results, having repurposed a dataset from a separate study [59]. We hypothesised that ML-based predictive models could be developed for the diagnosis of intractable veterinary diseases. In an earlier study, we investigated the feasibility of using ML to augment the diagnosis of non-effusive FIP, arguably one of the most difficult to diagnose diseases of cats [60]. The results from that preliminary study indicated that ensemble ML models detected nuanced patterns in a dataset of informative clinical and laboratory variables (or ‘features’ in ML parlance) to effectively discriminate non-effusive FIP cases from other diseases. The benefit of using ML over a classical diagnostic tree approach is the ability to run numerous scenarios over thousands of iterations within very short periods of time. The number of cases analysed, and the number of iterations performed in order to determine the patterns that predict the outcome, are too numerous to be feasibly analysed by a human, in a practical timeframe.

Effusions in cats are typically classified as either a transudate, a modified transudate or an exudate, based on a combination of the total protein measure and the total nucleated cell count (TNCC) [61,62]. Though not diagnostic for any condition, the classification of the effusion can steer the clinician towards differential diagnosis to explore. In cats the major differential diagnosis for pleural effusions are pyothorax/infectious pleuritis, effusive FIP, neoplasia, congestive heart failure (CHF), idiopathic chylothorax and a number of less frequently observed conditions [63]. Major differentials for abdominal effusions are FIP, lymphocytic cholangitis, cardiac disease (CD), neoplasia, bacterial peritonitis, renal or liver disease along with a number of rarer conditions [64].

In suspected cases of effusive FIP, fluid is routinely sampled. Aspiration of this fluid is a clinical procedure which is often performed for the welfare and comfort of the patient as well as the collection of valuable diagnostic samples [65]. Effusion samples are more easily and routinely collected than the samples required for diagnosis of non-effusive FIP cases, such as fine needle aspirates or biopsies which would require more invasive procedures with the stress of sampling potentially adversely affecting outcomes [25,45]. RT-qPCR of effusion has now been widely adopted in the field for the diagnosis of FIP, and in cases with a high index of suspicion of FIP, a positive result is regarded as de facto confirmation of disease [18,25]. Equally, a negative RT-qPCR result in a case with a low index of suspicion of FIP can be used to further discount a diagnosis of effusive FIP. Thus, over the last 20 years, the University of Glasgow Veterinary Diagnostic Service (VDS) has amassed a large dataset of effusion test results for which a substantial amount of outcome classification data, in machine learning terms the “ground truth”, is available. In machine learning analysis, a ground truth which is independent of the variables being modelled is the ideal scenario, as it reduces bias in the modelling. In order to undertake the present study, we needed to amass a sufficiently large number of FIP and non-FIP cases. Thus, in cats which possessed a high index of suspicion of FIP, based on European Advisory Board on Cat Diseases (ABCD) guidelines, we considered a positive RT-qPCR result as being diagnostic of FIP. Non-FIP cases were determined on the basis of an alternative diagnosis being made, and this was based on a variety of other diagnostic tests, or a negative RT-qPCR result substantiating a low index of suspicion. These additional tests included fluid cytology, bacterial culture and tissue histology; neither the RT-qPCR results nor the results from these auxiliary tests were included in the modelling exercise. The availability of this dataset facilitated the development of novel ML algorithms for the diagnosis of effusive FIP. Building on initial models constructed using solely signalment and laboratory markers to diagnose non-effusive disease [60], we have gone on to incorporate reported clinical signs into our ML methods in an attempt to increase prediction accuracy. Herein, we develop predictive diagnostic models to detect effusive FIP and we assess their performance. The ultimate goal of this work is to develop a decision-support tool for routine use in a clinical diagnostic laboratory.

## 2. Methods

### 2.1. Dataset and Data Preparation

The dataset used in this retrospective study was generated within the diagnostic laboratories of the VDS at the University of Glasgow. Samples were submitted as part of the normal diagnostic investigation of clinically unwell cats, with additional metadata being provided by the submitting clinicians. Samples were submitted between 2001 and 2025 from cats where there was a clinical suspicion of effusive FIP. Laboratory records, including laboratory measurements of routine and specialised biomarkers, supportive metadata (including signalment and clinical signs) and expert interpretation of the results generated, were extracted from the Laboratory Information Management System (LIMS). Cases were selected where samples had been submitted for a specific suite of fluid analysis tests tailored to provide the submitting practitioners with insightful data to aid in the diagnosis or exclusion of FIP. The profile included the following tests, all of which were performed on effusion samples: anti-FCoV serology, measurement of alpha-1-glycoprotein (AGP), fluid protein analysis and cytological smear analysis, with fluid differential cell counts also being recorded. Effusions originated mainly from the abdominal/peritoneal cavity (*n* = 467) and pleural/thoracic cavity (*n* = 247), with a low number submitted from the pericardium (*n* = 4).

Cases were selected for inclusion in the study if a complete set of laboratory results was available and signalment data was provided by the submitting clinician. In all cases analysed in this study, FIP was considered a differential diagnosis with a cavity effusion being one of the clinical signs reported. The level of detail provided regarding clinical signs varied and was missing in a proportion of cases. Test results and sample metadata were interpreted by VDS clinicians in line with ABCD FIP diagnostic guidelines [25], current at the time of interpretation. For data modelling purposes, outcome classification of cases was made based on additional testing including, but not limited to, RT-PCR, cytological examination, tissue histological examination and bacterial culture. In some cases these results were considered supportive of a presumptive diagnosis of FIP, while in others they indicated that FIP should be excluded as a differential diagnosis; in a proportion of non-FIP cases, these tests enabled an alternative diagnosis to be established.

Manual differential cell counts (DCC) were performed on each effusion sample where possible, and though not available for all cases, these were included in the modelling process for evaluation during feature selection. In a proportion of cases a differential cell count was not recorded, and in these cases only a commentary on the smear was recorded. DCC are supplied in the smear report as a percentage of the total nucleated cell count (TNCC; feature name “Fluid WBC”), where the cell lineage count represents greater than 1% of the TNCC. This percentage was used to estimate the differential cell count by multiplying the TNCC by the percentage stated for each cell lineage. The Fluid WBC count was stored as a numerical field in the LIMS; in the majority of cases, this was machine generated, and in a minority of instances this was generated by manual count (see Table 1 for DCC details).

All data were manually cleaned and curated into a format suitable for use in modelling, as described previously by Dunbar et al. [60]. Following this, the dataset was partitioned into three smaller datasets suitable for the steps required to train the models and for testing. A proportion of cases within the dataset included anatomical pathological findings and/or histopathology. These cases were held out of the dataset during partitioning so that they could be added to the “testing” data, as these cases were definitively diagnosed as having FIP or an alternative disease or cause of death was determined. The remaining cases were divided with 60%, balanced by outcome classification and selected for training; the remaining 40% were further subdivided with half, again balanced by outcome classification, allocated to the “Validation” partition, and the remaining half allocated to the “Testing” partition.

### 2.2. Exploratory Analysis

We conducted an extensive exploratory analysis, including examining reported clinical sign details, as these are potentially valuable data for modelling purposes. We used the clinical notes documented on the LIMS to extract keywords and phrases and evaluate their frequency within clinical notes, and these were included in the modelling as binary variables: present (1) or absent (0). Initial analysis was undertaken in “R” to generate a broad understanding of the data and determine patterns [66]. Correlated or co-varying features can be problematic for some algorithms, and for this reason correlations and co-variance were assessed using a correlation matrix, visually assessed with a correlation plot. Principal component analysis (PCA) was performed to assess the potential value of features within the dataset. Individual features were assessed for variation between disease classification groups and for statistically significant differences between groups. Density plots were generated for selected features and stratified by dataset and outcome in order to illustrate that the data shared across the training, validation and testing stages were representative of the overall dataset.

### 2.3. Feature Selection

Features are any datapoint used as an input variable in the modelling process. All laboratory data from the effusive FIP profile and derivatives such as effusion type, together with metadata such as signalment and effusion site, were prepared for inclusion in the models. Pre-existing knowledge was used to select commonly reported clinical signs associated with a diagnosis of FIP, and corresponding data was extracted from the supplied clinical histories. Outputs of iterative independent ensemble models were used in conjunction with exploratory analysis (detailed above) to assess feature importance. Due to constraints with the algorithms selected, we were unable to produce comparative feature importance measures, such as Gini index, for all base learners, therefore we opted for iterative removal of features to validate feature selection. Additionally, clinical pathologists provided expert guidance regarding redundant or less informative features.

### 2.4. Model Selection and Building

Building on our previous work, an ensemble model was developed that incorporated five differing types of classification algorithms each exploiting a different underlying methodology, either mathematical or statistical. In total 39 independent ensemble models were constructed with five classification algorithms used to build each ensemble model; these included Logistic regression (LR), Naïve Bayes classifier (NB), Support Vector Machine (SVM), extreme gradient boosting (XGBoost) and RandomForest (RF) [67,68,69,70]. Twenty-five base learners of each type were assembled, and the outputs of these models became the features of the ensemble model. The 39 models were built to allow iterative determination of the most informative features. The features available to the base learners differed in each independent ensemble model; models were systematically evaluated to select features for inclusion based on their contribution to performance.

Binary classification base learner models were trained using predictor variables listed in Table 1 (the list of features available to each ensemble are detailed in Supplementary Materials S3, feature list column) and one of two response variables corresponding to cases classified as either FIP or non FIP. A numerical matrix of data was used to train the models, as some algorithms included in the ensemble only accept numerical inputs. Data was centred and scaled during pre-processing. Laboratory data used as predictor variables were in a numerical format. A numeric “dummy binary variable” was generated for categorical variables including signalment and clinical signs using a method called “one-hot encoding” [69]. Response variables were similarly coded as a numeric “dummy binary variable” with cases classified as “non FIP” being coded as “0”, while “FIP” cased were coded as “1”.

Our ensemble model utilised a model based on the RandomForest algorithm to predict outcomes from an array of aggregated predictions generated from 125 base learner models. Base learner models utilised one of five underlying algorithms, either Logistic regression, Naïve Bayes classifier, Support Vector Machine, XGBoost or RandomForest. Data was down sampled during pre-processing to produce an evenly balanced dataset for training at each iteration of modelling. Each base learner produced an output prediction that was stored in an array and used as the input for the final RandomForest classifier.

The “caretEnsemble” package was used to assemble the base learner models, and the “caretList” function was used to build the batched models [69]. Base learner models were trained using ten-fold cross-validation, repeated ten times. This generated diversity among otherwise identical base learners. We used the “grid” function to automatically select optimal tuning parameters for the base learners; this function assesses accuracy and Cohens’s kappa (κ) on the cross-validation hold-out fraction. Additionally, these were assessed using the validation dataset. Tuning parameters differ between algorithms and, therefore, parameters differed between model types; optimised tuning grids are provided in Supplementary Material S1.

**Figure 1:**
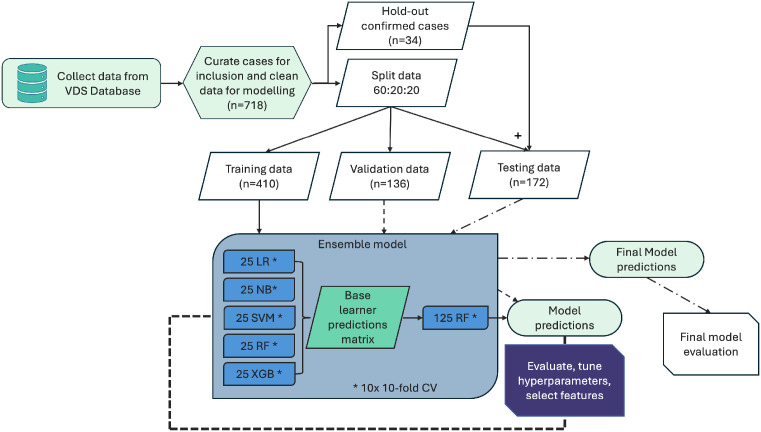
Modelling process overview. VDS = Veterinary Diagnostic Services, LR = Logistic regression, NB = Naïve Bayes, SVM = Support Vector Machine, RF = RandomForest, XGB = extreme gradient boosting, CV = cross validation.

The “caretStack” function from the “caretEnsemble” package was used to build the RandomForest ensemble model; similarly to the base learners, this used ten-fold cross-validation, repeated ten times. The ensemble RandomForest hyperparameter “mtry” was also optimised using a tuning grid which assessed “mtry” using integers between 40 and 50. This value represents the number of variables at each split during model building; the variables are selected at random at each node, and the “mtry” value was forced to a representative level in order to provide a proportionate selection of inputs for evaluation from the prediction array generated by the base learner models. The modelling process is detailed in Figure 1.

### 2.5. Model Evaluation

In addition to the manual partitions created for training, validation and testing, the models were trained using ten-fold cross-validation; a differing random seed for each base learner produced models with identical inputs and identical grids of hyperparameters but with differing data within the cross-validation folds. This allowed for nine folds to be used for training and the remaining fold to be used to evaluate the training performance. In addition to the cross-validation, the models were evaluated on the validation data, with the validation partition used to tune base learner models and select features. This was used to fine tune and evaluate changes in tuning parameters; a separate dataset for this step reduces information leakage and reduces bias in the models. Final evaluation was performed on the testing dataset, including a group of reference cases for which either pathology, histopathology and/or IHC had been performed to ascertain a definitive diagnosis of FIP or to determine an alternative diagnosis.

Confusion matrices were generated to evaluate model performance using the model predictions from the ensemble model and the reference outcome classification as described above. We used the same performance metrics to evaluate the ML models as we would use to evaluate a newly developed diagnostic tool, including accuracy, sensitivity, specificity and inter-rater agreement (Cohen’s kappa, κ), and these were used to assess each dataset. We assessed model performance based on completely unseen data in order avoid introducing any bias to the metrics and to mimic the use of models on real world data. Area under the receiver operator curve (AUC) was also calculated. For all analyses included in this study an alpha level (*p*-value) < 0.05 was considered statistically significant.

### 2.6. Software and Model Development Environments

The statistical programming language “R” (version 4.1.2) [66] was used throughout for data preparation, data cleaning, modelling and output of results. The following packages were used: “Base R” and “stats” (both version 4.1.2), “Tidyverse” (version 2.0.0) for data manipulation and data visualisation. Exploratory analysis utilised the “cor”, “pairs” and “prcomp” functions from “Base” for the analysis of co-variance, correlation and PCA. “Caret” (version 6.0-90) and “CaretEnsemble” (version 2.0.1) were used for modelling, including pre-processing, algorithm functions, ensemble model building, training, model evaluation and metric generation. “pROC” (version 1.18.0) was used to generate the AUC values. A list of all packages loaded during processing is noted in Supplementary Material S2.

## 3. Results

### 3.1. Case Summary

A total of 718 cases submitted with a clinical suspicion of effusive FIP, that had a complete set of laboratory markers, signalment and a diagnosis, were available from the LIMS. Among these, pathology and histopathology data was available for 24 cases for which a diagnosis of FIP was made and 10 cases for which an alternative diagnosis was made. The remaining cases were classified as follows: 312 cases of FIP based on a supportive PCR, 313 cases where an alternative diagnosis was determined by cytological findings and 59 cases where a negative PCR result combined with other laboratory data clearly ruled out FIP. Cases where the clinical notes stated a patient was already undergoing anti-viral treatment for FIP were excluded.

In 65% (*n* = 467) of suspected FIP cases, the animals presented with an abdominal effusion, sometimes alternatively described as ‘ascites’ or a ‘peritoneal effusion’. Pleural or thoracic effusion was described in 34.4% (*n* = 247) of presentations, and only 0.6% (*n* = 4) of presentations involved a pericardial effusion. Effusions were classified as transudates in 3.6% (*n* = 26) of samples, as modified transudates in 49.2% (*n* = 353) and as exudates in 47.2% (*n* = 339). At least one clinical sign was noted on 75.8% of submissions (544 of 718); within these the frequency of description of clinical signs was as follows: lethargy 21.1% (*n* = 115), pyrexia 25.9% (*n* = 141), icterus 5.3% (*n* = 29), anorexia 25% (*n* = 136), inappetence 19.9% (*n* = 108), dyspnoea 10.3% (*n* = 56), diarrhoea 5.5% (*n* = 30), pallor 3.9% (*n* = 21), vomiting 3.5% (*n* = 19) and a mass was described in 2.9% (*n* = 16) of submissions. Other metadata was noted, such as retroviral screening results; however, there were insufficient numbers to merit inclusion in the analysis.

### 3.2. Exploratory Analysis

**Figure 2:**
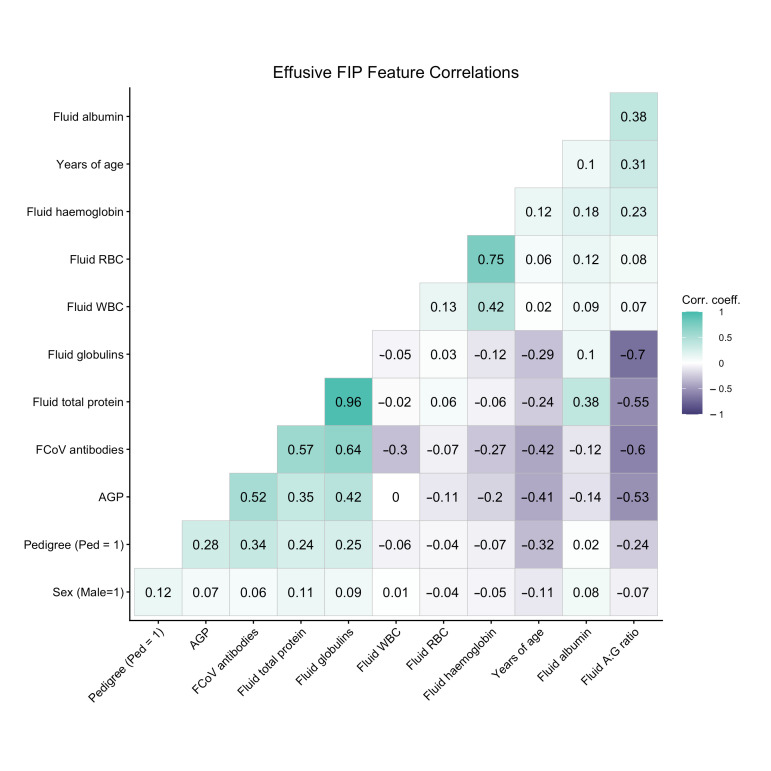
Correlation of features. This is a visualisation of the correlations within the major contributing laboratory markers; the value in the box is the Pearson correlation coefficient.

**Figure 3:**
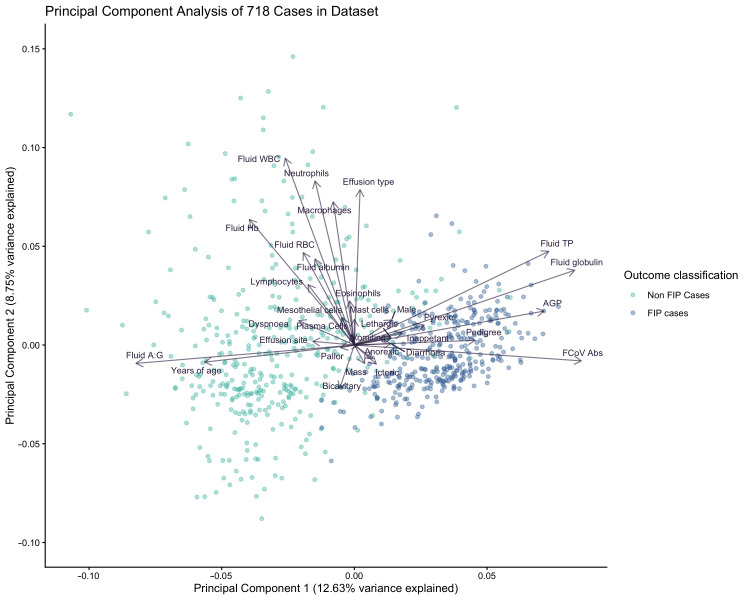
Principal component analysis (PCA) of all 718 cases in the study. Separation is evident in just these first two principal components (PC). A total of 21.38% of the variance in the data is explained by the first two PC, with 12.63% explained by PC1, and 8.75% explained by PC2.

**Figure 4:**
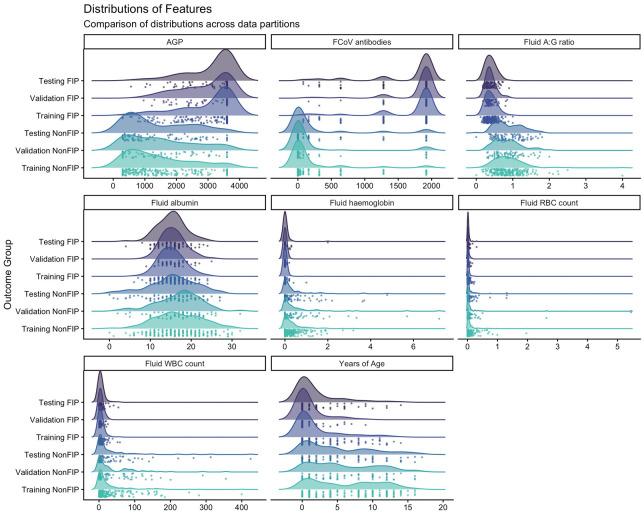
Density plots of the continuous laboratory markers, stratified by data group (training, validation or testing) and by outcome classification (FIP or non-FIP disease). Below the density plots the rainfall scatter shows the distribution of individual points contributing to the density plot.

We assessed correlation and covariance of signalment and laboratory data features using Pearsons correlations (Figure 2). As one would expect, correlations are evident between linked markers, for example, between total protein and immunoglobulin and between haemoglobin and erythrocyte counts. There was a moderate correlation between FCoV antibodies and AGP, and both of these markers show mild or moderate correlation with pedigree and age, respectively. We scrutinised the impact of features on separation of FIP cases and controls in principal component analysis (Figure 3) and looked at the contributions each variable made by evaluating the loading vectors. The PCA demonstrates that increased FCoV titre, AGP, TP and specifically globulins are strong indicators for FIP. Commonly cited signalment and clinical signs of FIP including sex, pedigree, lethargy, inappetence, icterus, pyrexia and diarrhoea also were found to aid in separation of FIP cases from those of other diseases, although to a lesser extent. Meanwhile, advancing age, a high A:G ratio and cellular markers appear to influence clustering of non-FIP disease. Some of the insights gained here were used in the feature selection for modelling. Additionally, we looked at the individual features and assessed the differences in these between outcome groups using density plots (Figure 4); these illustrate that each data partition was representative of the overall dataset.

### 3.3. Data Partitions

The overall dataset was divided into training, validation and testing data in order to reduce information leakage and model bias from overfitting to a single dataset. As described in the methods, cases for which pathology/histopathology was available (*n* = 34) were held out to be incorporated into the testing data partition. The remaining data (*n* = 684) was subdivided into three subsets, generated by partitioning according to the same protocol. The training data constituted 60% of that remaining dataset (*n* = 410). The other 40% was further divided to produce the validation data (*n* = 136) and test data (*n* = 138). The final testing dataset was produced by adding the 34 pathology cases, giving a total of 172 cases for final evaluation.

### 3.4. Feature Selection

The original data download from the LIMS included all of the signalment, clinical notes, laboratory test results, smear reports and clinical interpretations. These data were cleaned to produce 32 features for evaluation in modelling. The curated feature list consisted of 9 laboratory markers, 3 signalment features, 10 clinical signs, 7 differential cell counts and 3 effusion descriptors. A full list of features selected for use in the modelling, including those dropped from the final model, are described in Table 1. Using a combination of a priori feature selection informed by the exploratory analysis and clinical knowledge, the features included in the final model were identified as worthy contributors based on iterative inclusion and exclusion from the model.

The best performing model incorporated 6 laboratory markers, 3 signalment elements, all clinical signs (*n* = 10) and the ‘site of effusion’. Fluid total protein (TP), Fluid globulin and Fluid RBC were not included as they did not provide any additional information to the model; in some iterations their inclusion resulted in a performance reduction. These three measurements were among the correlated variables: globulin is a derivative of TP, and these correlate with each other and with A:G ratio. TP also correlates with Fluid albumin, while Fluid RBC correlates highly with Fluid haemoglobin. These correlations are illustrated in the correlation plot in Figure 2.

### 3.5. Model Performance and Statistical Analysis

**Figure 5:**
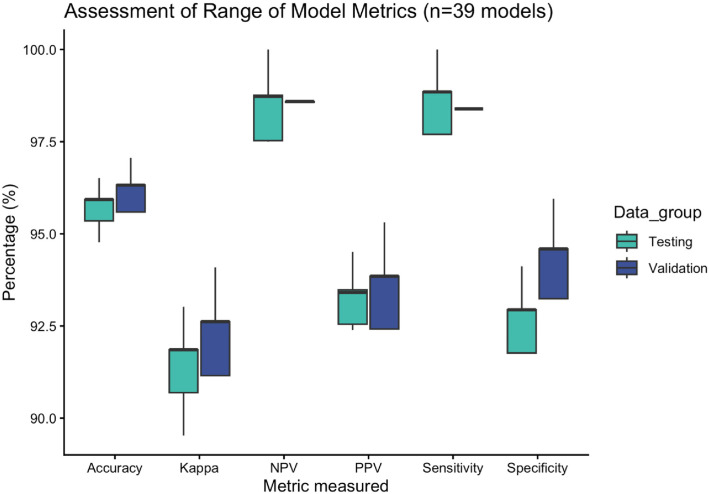
Range of Model metric—Range of Accuracy, Cohen’s kappa, negative predictive value (NPV), positive predictive value (PPV), sensitivity and specificity from the 39 iteratively build models are shown. Performance measures of each model on both the validation and training data are illustrated; testing in green, validation in blue.

All 39 ensemble models showed a high predictive accuracy when compared to the ground truth. A summary of metrics from all 39 models is shown in Figure 5, and an overview of the metrics from the five top-performing models is shown in Table 2 (model metrics from all 39 models as shown in Supplementary Materials S3–S5). The range of accuracy on the validation data was 95.59–97.06%, and the range of accuracy on the testing data was 94.77–96.51%. The Cohen’s kappa (k), a measure of inter-rater agreement, also produced high measures of agreement in the range 91.15–94.09% and 89.53–93.02% on the validation and testing data, respectively. Sensitivity and specificity are two further measures of performance often used to evaluate diagnostic tests. The range of sensitivity was 98.39–98.39% on the validation data, 93.24–95.95% on the test data, and for specificity 97.7–100% and 91.76–94.12%, respectively. The range of the mean resample accuracies from the RandomForest ensemble was 92.68–95.0% (See Figure 5 and Table 2)

The best performing model comprised a total of 20 features, including laboratory markers (minus TP, Fluid RBC and Fluid globulin), all signalment, all clinical signs and effusion site. This model had a predictive accuracy of 96.32% (95% CI 91.63–98.80), Cohen’s kappa of 92.62%, sensitivity 98.39% and specificity of 94.59% on the validation data. It had a predictive accuracy of 96.51% (95% CI 92.56–98.71), Cohen’s kappa of 93.02%, sensitivity 98.85% and specificity of 94.12% on the test data. The best performer was also evaluated by assessing the area under the curve (AUC); on the validation data this was 96.49% (95% CI 93.45–99.53), and on the test data this was 96.48% (95% CI 93.73–99.24). Confusion matrices for the best performing model are presented in Table 3 and Table 4 for validation data and testing data, respectively. The model incorrectly predicted disease status in five individuals within the validation dataset (four false positives, one false negative) and in six individuals in the testing dataset (five false positives, one false negative).

## 4. Discussion

The aim of this study was to expand upon the development of predictive diagnostic models for FIP. Using the clinical data from effusive cases where supportive evidence of an FIP diagnosis or alternative diagnosis is arguably easier to achieve provides us with a dataset where we have more confidence in the “ground truth”. We previously illustrated, using non-effusive FIP as an example, that clinical datasets lend themselves to the creation of predictive models. The PCA (Figure 3) shows near distinct clusters of disease groups, with few examples of overlap. There are clearly mathematically discernible patterns underlying these data, and we believe ML can exploit these in a quantitative manner, thus avoiding some of the subjectivity of human interpretation. The patterns observed in the clinical findings for this disease reflect the inflammatory nature of FIP. Rather than being focused on haematology and blood biochemistry, the laboratory work-up for a suspected case of effusive FIP is very often primarily focused on direct analysis of the effusion.

Transudates are most commonly associated with a handful of systemic conditions, namely CHF, liver or renal disease and are associated with increased hydrostatic pressure and reduced osmotic pressure. In contrast, exudates and many modified transudates are formed as a result of increasing permeability of tissue capillaries, allowing the passage of protein and cellular material into body cavities. This permeability arises from numerous conditions such as neoplasia, inflammatory disease, infectious disease and a number of other disease processes. Of the samples included in this study, there was a near equal balance of modified transudates and exudates, 49.2% and 47.2%, respectively, with transudates representing only 3.6% of samples. Other studies suggest findings from 4.76% [71] to 24% [72]; however, the setting in which the data is collected plays a role, and in both of these examples the case series arose from a referral veterinary hospital, where availability of specialist care may influence the cases presented. Although observations may be an under-representation, transudates are relatively uncommon in cats [63]. Additionally, the gross appearance and typical aetiology of transudates possibly reduces the likelihood that these would be submitted for effusive FIP testing, as the initial in-clinic analysis would markedly reduce the suspicion of FIP and steer investigations in an alternate direction, and therefore our sample set may have been biased against transudates. The proportions observed for the site of effusion are similar to those described elsewhere [71]. Studies often focus on either pleural effusion or abdominal effusions, with a few covering bicavitary effusions. FIP is typically among the list of diseases diagnosed for effusions regardless of site [63,64,71,73,74,75,76]. In several of these studies FIP more commonly presents with abdominal effusion than pleural effusion, where cardiovascular disease, neoplasia and pyothorax are more frequently described [71].

The incorporation of clinical signs is an advance on merely training models on signalment and numerical laboratory markers. Clinical signs of FIP are non-specific, and although they may provide evidence to support a suspicion of FIP, they could equally be attributable to a number of alternative conditions. We manually scored the top ten clinical signs noted within clinical histories supplied with submission of the original diagnostic samples; these were scored for presence of a clinical sign, the absence of the sign or if the sign was omitted or missing. We could have used imputation to estimate the rate of these clinical signs in the cases where none were noted; however, we elected to score these as “not present” rather than add estimates. In a previous study of 82 cats with pleural effusion, 18% had FIP, and clinical history was provided for 73 of these cats. Clinical signs included dyspnoea/tachypnoea in 69%, lethargy in 36%, inappetence and/or weight loss in 27%, pyrexia in 3% and icterus in 1%; other signs listed included dysphagia/regurgitation, cough, cyanosis, acute collapse, cool extremities and haemoptysis with frequencies descending from 12 to 3%, respectively [73]. Another study found cats with pericardial effusions reported that 59% of cases had tachypnoea, 2% reported hyperthermia and 30% reported hypothermia [75]. In a study summarising findings in 42 confirmed cases of FIP, the following clinical signs were noted: inappetence in 96% of cases, weight loss in 88%, pale mucous membranes in 68%, fever in 63%, palpable abdominal mass in 48%, ascites in 45%, gastrointestinal signs in 36% and dyspnoea and jaundice in 29% and 26% of cases, respectively [27]. Additional signs included neurological and ocular signs, abdominal pain and palpable sub-mandibular lymph nodes. An effusion was reported in only 45% of these cases; this data is therefore not directly comparable to our dataset, as non-effusive cases were included. However, there is significant overlap in general clinical signs across the spectrum of FIP presentations, and we have incorporated the major clinical signs into our modelling efforts.

In contrast to our previous non-effusive ML study in which we relied upon clinical interpretation of the laboratory markers, signalment and clinical signs alone as the outcome classifier for ground truth [60], in this study cases were classified using both expert interpretation as well as an additional supportive test or diagnostic identification of an alternative cause of clinical signs, providing a substantially supported ground truth. Our aim was to progress beyond feasibility to demonstrate that, using a curated and independent dataset, ML can provide highly accurate predictions of disease status of cats presenting with a cavitary effusion and a clinical suspicion of FIP. It was somewhat inevitable that the performance metrics on this smaller independent dataset were slightly poorer compared to models trained solely on expert opinion cases; however, the models exhibit less bias and are more robust, and performance was reduced only marginally. If these models were being evaluated as novel diagnostic tests, these performance metrics would be considered highly reliable. The accuracy, sensitivity and specificity of the top-performing five models was identical, and the resample accuracies of the ensemble were used to determine the final model. Moreover, the performance of all 39 iterative model builds was high, and so numerous models may be considered viable options. The performance data from the five best-performing models are shown in Table 2, illustrating the various combinations of features which would all provide a highly accurate model. It is possible that data with fewer sparse fields in the clinical notes would improve model performance further and could lead to a slightly different feature set. However, the performance of the models on this current dataset reflects the data collected by a veterinary referral laboratory, where often clinical histories and follow-up are incomplete, and thus our samples represent a ‘snapshot’ in time, and details provided are at the mercy of the referring clinicians.

FIP is an immune-mediated disease with a viral aetiology; therefore, the markers used to assess the extent of inflammation and consequently to diagnose disease are generally the same markers that are used for numerous other infectious and non-infectious diseases. Indeed, the non-specific nature of these markers is one of the challenges of diagnosing FIP. Treatment using specific anti-viral therapy has been shown to rapidly reduce clinical signs associated with FIP, and it is therefore likely to rapidly alter the inflammatory biomarker profiles in the bloodstream and the composition of effusions [77,78,79]. For this reason, we elected to exclude cases known to be undergoing or have had anti-viral treatment, and only cases submitted for initial diagnosis were included in the study.

Interrogating the samples that were misclassified by our models illustrates the problem of marker non-specificity and commonality with other disease processes. The model misclassified five cases from the validation dataset. One of these cases, a putative false negative result, was designated as FIP positive on the basis of RT-qPCR. However, the A:G ratio was relatively high compared with most FIP cases. This cat was alive and well eight months post-testing and, as this was prior to the availability of curative treatment, the veracity of the FIP diagnosis is doubtful. Of the four false positive cases, one case had a profile which was somewhat suggestive of FIP, however the A:G ratio was relatively high, and the age of the animal and the appearance of the effusion was not typical for FIP. Nevertheless, the AGP and FCoV titres were high, as was the protein, and we detected no virus in the effusion; these results concurred with results from previous testing undertaken elsewhere. Three further cases that were diagnosed as bacterial peritonitis (classified on the basis of cytology, which revealed septic inflammation with intra- and extra-cellular bacteria) were predicted to have FIP by the model. All three showed high AGP, low A:G ratios and moderate or high antibody titres. It is possible that the FCoV serology results could have been incidental findings, as all three were pedigree cats, which have an increased risk of exposure. Additionally, although the diagnosis of bacterial peritonitis was made, in any of these three cases there is the possibility of an underlying FIP, although possibly FIP was less likely in one case, a sudden death. The model misclassified a total of six cases from the testing dataset. This included one false negative from a case with a profile suggestive of FIP, which was supported by a positive RT-qPCR. This case had an A:G ratio of 0.73, which might have contributed to the misclassification, as this is a higher value than that observed typically in cases of FIP. A total of five false positives included two further septic exudates in which no virus was detected by RT-qPCR, and it is possible an underlying FIP may also have been present in these cases. One false positive case was determined to be epithelial neoplasia, while in two cases the effusions were found to be transudates. This type of effusion, not classically associated with FIP, was under-represented in this dataset, and thus the model may not have been able to associate low protein fluid as being a signal of ‘not FIP’. The high FCoV titre and low A:G ratios most likely accounted for the misclassification of these two cases, as the AGP was normal in one and only mildly elevated in the other. Unfortunately, client cost constraints often prohibit extensive investigation, additional testing is not always performed and follow-up submissions are rare. The lack of the complete clinical records is an inevitable constraint to the type of retrospective study we have undertaken.

The samples analysed represent a ‘snapshot in time’, and therefore the stage of disease was variable among the cases. Non-effusive FIP is often insidious and similar to some neoplastic diseases, whereas effusive FIP and bacterial infections show more rapid onset. Clinical presentations can be highly variable; each is unique, and the pathogenesis can vary depending on specific stressors for the infected or diseased host. Furthermore, the development of disease in cases of FIP, as well as other infectious and non-infectious diseases, can be acute or chronic, and samples can be sent for diagnosis either at the early stage of infection when clinical signs first appear, or later, when disease is more fulminant. The generation of larger databases of confirmed cases will provide an opportunity to build better models, using cohorts of cases at various stages of disease.

This dataset is typical of many LIMS-derived datasets, which often contain sparse fields. Laboratory features and signalment were available for most cases, whereas clinical signs and differential cell counts were often not available, these being features where data is likely to be sparse. In general, clinical signs might not be present in some cases, or they might not be reported in others. The variable nature of effusion samples can affect their suitability for differential cell count testing, and effusions that did not have machine or manual leukocyte counts performed could not be included in this study. Furthermore, a number of factors determine whether cell counts can be performed; suitability of the effusion to be passed through an analyser determines if a machine count is possible, and manual counts are generally dependent on the quality of the smear. Smear quality can be affected by the preparation of the smear, time since sampling and degradation of the sample. Additionally, manual counts can be affected by intra- or inter-operator variability. These sparse matrix features were assessed during the development and evaluation of the models and were included to maximise information gain. As missing data can be problematic for some algorithms, a dummy variable “0” was imputed if data were missing. This compromise enabled the inclusion of data in the sparse matrix without introducing bias.

The question of sparse data for the differential cell counts later became irrelevant, as they were not included in the final model. After assessing various inclusion patterns, it was evident that these did not particularly help to discriminate between disease states, and so these (as well as correlated variables, ‘effusion type’ and ‘bicavitary status’) were removed as they were redundant. Including the type of effusion did not improve discrimination, likely as this feature is a derivative of the protein and cellular content of the effusion, and this information is already included in other features. Similarly, whether an effusion was bicavitary might allow discrimination between non-FIP diseases, but did not help to discriminate between FIP versus non-FIP. The Pedigree category was another example of sparse data. As the numbers of some breeds were much lower compared to others that were over-represented, we elected to maintain Pedigree as a single grouped feature. This proved to be a valuable feature, ranking typically in the top ten features in both this and the previous study. Specific breeds have in the past been associated with increased risk of developing FIP, and this is widened to include pedigree cats in general as being pre-disposed, in most guidance [25,80,81].

It is our belief that a clinical understanding of the disease and disease processes are prerequisites for any team who embark on developing diagnostic tools using either machine learning or any other form of artificial intelligence. We base this assertion on our previous work, where we found FCoV titre and AGP to be highly predictive of FIP using an extreme gradient boosting method, yet our clinical understanding cautions against reliance on these markers to the exclusion of all others [60]. Clinical signs in the absence of any laboratory markers were modelled in one study, and [58] given that clinical signs of FIP are non-specific and shared with numerous conditions, this seems to present a less than optimal diagnostic approach. Two other studies relied heavily on RT-qPCR: one assessed a myriad of tissues, which in a living patient is impractical [59]; the other concluded that analysis of effusion or blood were the best tests to use for FIP diagnosis [57]. Whilst the literature is supportive of RT-qPCR use as a tool to support a presumptive diagnosis, there is no definitive, standalone molecular test for FIP. Additionally, there is evidence to suggest that detection of FCoV in blood is not helpful in identifying FIP [33,38]. In a disease as complex as FIP, a carefully curated dataset and strong clinical understanding of the disease is a key component of the modelling process.

Designing a tool which is robust to all the variability of diagnostic testing in different countries is a significant task. While guidelines have been published in Europe [25] and the United States [51], their adoption is inconsistent worldwide. The diversity of available tests and lack of standard protocols mean that test results might not be directly comparable. FCoV serology is an example of a test which is widely available, but not necessarily widely adopted; in addition, there is significant variability amongst methods, both in terms of style of test and range of analysis. As a degree of standardisation is essential to generate representative results that can be interpreted by a ML model, the development and adoption of a universal ML model to diagnose FIP is an extremely challenging prospect.

## 5. Conclusions

We have demonstrated that highly accurate predictive machine models based on laboratory datasets can be developed for effusive FIP, in a similar way to those we have developed for the non-effusive form of the disease. We have further shown that incorporating clinical observations at the time of sampling can provide the most accurate diagnostic predictions. This finding underscores the importance of practitioners providing relevant clinical history when submitting samples to diagnostic laboratories to maximise the quality of result interpretation. Our results demonstrate that there is clear potential for machine learning based decision support systems to be created for use in specialist diagnostic laboratories. A tool such as this could be used to standardise interpretation of typical laboratory-generated data. However, a number of challenges need to be overcome before this becomes a reality, including issues of inter-laboratory variation in test methodology and data harmonisation. With the advent of curative treatment for FIP and the option for presumptive diagnosis based on response to treatment, it may be argued that proportionately fewer cats may be subjected to full diagnostic investigation, making the collection of future FIP case series data more challenging. Nevertheless, this study represents another step towards the goal of generating a useable tool for FIP diagnostics that can be deployed in a veterinary laboratory setting.

## Figures and Tables

**Table 1:** Feature List, including summary statistics stratified by dataset, either training, validation or testing and disease classification, either non-FIP disease or FIP.

			Training		Validation		Testing		Overall	
Feature Group	Feature Name	Method	Non-FIP (*n* = 223)	FIP (*n* = 187)	Non-FIP (*n* = 74)	FIP (*n* = 62)	Non-FIP *(n* = 85)	FIP (*n* = 87)	Non-FIP (*n* = 382)	FIP (*n* = 336)
Serology	FCoV titre	Immunofluorescence ^◊^								
	Median [Min, Max]		0 [0, 1920]	1920 [80.0, 1920]	0 [0, 1920]	1920 [160, 1920]	0 [0, 1920]	1920 [80.0, 1920]	0 [0, 1920]	1920 [80.0, 1920]
Cytology	Fluid RBC count (×10^12^/L)	Siemens Advia 120 analyser ^†^								
	Mean [Min, Max]		0.0894 [0, 1.96]	0.0282 [0, 0.230]	0.17 [0, 5.45]	0.026 [0, 0.160]	0.097 [0, 1.31]	0.0252 [0, 0.300]	0.107 [0, 5.45]	0.027 [0, 0.300]
	Fluid haemoglobin (g/dL)	Siemens Advia 120 analyser ^†^								
	Mean [Min, Max]		0.347 [0, 3.19]	0.0134 [0, 1.00]	0.62 [0, 7.20]	0.008 [0, 0.200]	0.459 [0, 4.77]	0.0393 [0, 2.00]	0.425 [0, 7.20]	0.019 [0, 2.00]
	Fluid WBC count (×10^9^/L)	Siemens Advia 120 analyser ^†^/manual count								
	Mean [Min, Max]		30.1 [0.0300, 400]	5.47 [0.110, 58.5]	45.3 [0.170, 356]	5.09 [0.320, 24.9]	36.8 [0.0800, 425]	5.65 [0.200, 54.2]	34.6 [0.030, 425]	5.45 [0.110, 58.5]
	Lymphocyte count (×10^9^/L)	Manual cell count								
	Mean [Min, Max]		1.39 [0, 52.2]	0.243 [0, 5.17]	0.916 [0, 18.2]	0.25 [0, 2.25]	1.16 [0, 20.5]	0.295 [0, 3.25]	1.25 [0, 52.2]	0.258 [0, 5.17]
	Neutrophil count (×10^9^/L)	Manual cell count								
	Mean [Min, Max]		12.1 [0, 376]	3.66 [0, 49.1]	24.9 [0, 338]	3.39 [0, 18.5]	14.2 [0, 209]	3.83 [0, 41.2]	15.1 [0, 376]	3.65 [0, 49.1]
	Macrophage count (×10^9^/L)	Manual cell count								
	Mean [Min, Max]		1.18 [0, 51.9]	0.709 [0, 8.78]	2.11 [0, 58.1]	0.604 [0, 5.52]	1.44 [0, 34.8]	0.833 [0, 9.76]	1.42 [0, 58.1]	0.722 0, 9.76]
	Eosinophil count (×10^9^/L)	Manual cell count								
	Mean [Min, Max]		0.0477 [0, 2.83]	0.0043 [0, 0.283]	0.0129 [0, 0.270]	0.0052 [0, 0.251]	3.56 [0, 297]	0.002 [0, 0.0988]	0.822 [0, 297]	0.004 [0, 0.283]
	Plasma cell count	Manual cell count								
	Mean [Min, Max]		0.0016 [0, 0.357]	0.0016 [0, 0.283]	0 [0, 0]	0.00087 [0, 0.0364]	0.0002 [0, 0.0174]	0 [0, 0]	0.001 [0, 0.357]	0.001 [0, 0.283]
	Mast cell count (×10^9^/L)	Manual cell count								
	Mean [Min, Max]		0.0056 [0, 1.23]	0.0026 [0, 0.283]	0.0108 [0, 0.786]	0.0021 [0, 0.120]	0.0031 [0, 0.226]	0.0714 [0, 0.301]	0.006 [0, 1.23]	0.004 [0, 0.301]
	Mesothelial cells (×10^9^/L)	Manual cell count								
	Mean [Min, Max]		0.0005 [0, 0.0712]	0 [0, 0]	0.0031 [0, 0.222]	0.0001 [0, 0.0044]	0.0069 [0, 0.590]	0 [0, 0]	0.002 [0, 0.590]	0.00001 [0, 0.004]
Biochemistry	Fluid total protein (g/L)	Siemens Dimension Xpand Plus analyser								
	Mean [Min, Max]		39.6 [1.00, 110]	56.5 [28.0, 109]	40.6 [4.00, 109]	57.6 [26.0, 88.0]	34.9 [1.00, 69.0]	56.7 [18.0, 94.0]	38.8 [1.00, 110]	56.8 [18.0, 109]
	Fluid albumin (g/L )	Siemens Dimension Xpand Plus analyser								
	Mean [Min, Max]		16.9 [0, 32.0]	15.1 [6.00, 24.0]	17.0 [2.00, 32.0]	15.6 [9.00, 25.0]	15.4 [0, 27.0]	15.2 [4.00, 24.0]	16.6 [0, 32.0]	15.2 [4.00, 25.0]
	Fluid globulins (g/L)	Siemens Dimension Xpand Plus analyser								
	Mean [Min, Max]		22.7 [1.00, 87.0]	41.4 [16.0, 97.0]	23.6 [2.00, 87.0]	42.0 [17.0, 65.0]	19.5 [1.00, 48.0]	41.6 [14.0, 81.0]	22.1 1.00, 87.0]	41.5 [14.0, 97.0]
	A:G ratio	Siemens Dimension Xpand Plus analyser								
	Median [Min, Max]		0.820 [0, 4.00]	0.380 [0.120, 1.00]	0.840 [0.300, 2.00]	0.360 [0.180, 0.850]	0.800 [0, 1.80]	0.390 [0.180, 0.900]	0.820 [0, 4.00]	0.380 [0.120, 1.00]
	AGP (µg/mL)	RID */ELISA								
	Mean [Min, Max]		1440 [299, 3600]	2970 [299, 3600]	1540 [299, 3600]	2940 [960, 3600]	1440 [299, 3600]	3100 [560, 3600]	1460 [299, 3600]	3000 [299, 3600]
Signalment	Age (years)									
	Mean [Min, Max]		6.26 [0, 16.0]	2.07 [0, 14.0]	5.96 [0, 16.0]	0.968 [0, 9.00]	5.06 [0, 17.0]	1.97 [0, 14.0]	5.93 [0, 17.0]	1.84 [0, 14.0]
	Sex *n* (%)									
	Male		114 (51%)	119 (64%)	43 (58%)	43 (69%)	51 (60%)	59 (68%)	208 (54%)	221 (66%)
	Female		109 (49%)	68 (36%)	31 (42%)	19 (31%)	34 (40%)	28 (32%)	174 (46%)	115 (34%)
	Pedigree n (%)									
	Pedigree		44 (20%)	106 (57%)	18 (24%)	41 (66%)	24 (28%)	51 (59%)	86 (23%)	198 (59%)
	Not Pedigree		179 (80%)	81 (43%)	56 (76%)	21 (34%)	61 (72%)	36 (41%)	296 (77%)	138 (41%)
Effusion Information	Bicavitary effusion *n* (%)									
	Present		7 (3%)	1 (1%)	3 (4%)	1 (2%)	1 (1%)	2 (2%)	11 (3%)	4 (1%)
	Absent		216 (97%)	186 (99%)	71 (96%)	61 (98%)	84 (99%)	85 (98%)	371 (97%)	332 (99%)
	Effusion type n (%)									
	Transudate		10 (4%)	1 (1%)	4 (5%)	0 (0%)	11 (13%)	0 (0%)	25 (7%)	1 (0%)
	Modified Transudate		87 (39%)	114 (61%)	28 (38%)	39 (63%)	29 (34%)	56 (64%)	144 (38%)	209 (62%)
	Exudate		126 (57%)	72 (39%)	42 (57%)	23 (37%)	45 (53%)	31 (36%)	213 (56%)	126 (38%)
	Effusion site *n* (%)									
	Ascites		85 (38%)	116 (62%)	36 (49%)	42 (68%)	38 (45%)	57 (66%)	159 (42%)	215 (64%)
	Pleural		114 (51%)	41 (22%)	35 (47%)	8 (13%)	36 (42%)	11 (13%)	185 (48%)	60 (18%)
	Peritoneal		22 (10%)	29 (16%)	3 (4%)	12 (19%)	8 (9%)	19 (22%)	33 (9%)	60 (18%)
	Thoracic		1 (0%)	0 (0%)	0 (0%)	0 (0%)	1 (1%)	0 (0%)	2 (1%)	0 (0%)
	Pericardial		1 (0%)	1 (1%)	0 (0%)	0 (0%)	2 (2%)	0 (0%)	3 (1%)	1 (0%)
Clinical signs	Lethargy n (%)									
	Present		31 (14%)	38 (20%)	11 (15%)	8 (13%)	8 (9%)	19 (22%)	50 (13%)	65 (19%)
	Absent		192 (86%)	149 (80%)	63 (85%)	54 (87%)	77 (91%)	68 (78%)	332 (87%)	271 (81%)
	Icterus *n* (%)									
	Present		7 (3%)	9 (5%)	2 (3%)	4 (6%)	1 1%)	6 (7%)	10 (3%)	19 (6%)
	Absent		216 (97%)	178 (95%)	72 (97%)	58 (94%)	84 (99%)	81 (93%)	372 (97%)	317 (94%)
	Pyrexia *n* (%)									
	Present		28 (13%)	55 (29%)	11 (15%)	12 (19%)	12 (14%)	23 (26%)	51 (13%)	90 (27%)
	Absent		195 (87%)	132 (71%)	63 (85%)	50 (81%)	73 (86%)	64 (74%)	331 (87%)	246 (73%)
	Anorexia *n* (%)									
	Present		37 (17%)	46 (25%)	14 (19%)	11 (18%)	13 (15%)	15 (17%)	64 (17%)	72 (21%)
	Absent		186 (83%)	141 (75%)	60 (81%)	51 (82%)	72 (85%)	72 (83%)	318 (83%)	264 (79%)
	Inappetence n (%)									
	Present		31 (14%)	33 (18%)	9 (12%)	10 (16%)	8 (9%)	17 (20%)	48 (13%)	60 (18%)
	Absent		192 (86%)	154 (82%)	65 (88%)	52 (84%)	77 (91%)	70 (80%)	334 (87%)	276 (82%)
	Dyspnoea *n* (%)									
	Present		25 (11%)	10 (5%)	8 (11%)	1 (2%)	10 (12%)	2 (2%)	43 (11%)	13 (4%)
	Absent		198 (89%)	177 (95%)	66 (89%)	61 (98%)	75 (88%)	85 (98%)	339 (89%)	323 (96%)
	Diarrhoea n (%)									
	Present		6 (3%)	14 (7%)	1 (1%)	3 (5%)	3 (4%)	3 (3%)	10 (3%)	20 (6%)
	Absent		217 (97%)	173 (93%)	73 (99%)	59 (95%)	82 (96%)	84 (97%)	372 (97%)	316 (94%)
	Pallor *n* (%)									
	Present		9 (4%)	6 (3%)	2 (3%)	1 (2%)	2 (2%)	1 (1%)	13 (3%)	8 (2%)
	Absent		214 (96%)	181 (97%)	72 (97%)	61 (98%)	83 (98%)	86 (99%)	369 (97%)	328 (98%)
	Vomiting *n* (%)									
	Present		5 (2%)	4 (2%)	2 (3%)	2 (3%)	4 (5%)	2 (2%)	11 (3%)	8 (2%)
	Absent		218 (98%)	183 (98%)	72 (97%)	60 (97%)	81 (95%)	85 (98%)	371 (97%)	328 (98%)
	Mass *n* (%)									
	Present		5 (2%)	7 (4%)	0 (0%)	0 (0%)	2 (2%)	2 (2%)	7 (2%)	9 (3%)
	Absent		218 (98%)	180 (96%)	74 (100%)	62 (100%)	83 (98%)	85 (98%)	375 (98%)	327 (97%)

**^◊^** Titres recorded as >1280 were replaced with a half dilution above, i.e., 1920, for the purposes of modelling. * RID was retired and replaced with ELISA due to availability of reagents. ^†^ Sysmex XN-V analyser replaced Siemens Advia 120 from 2022. Advia and Siemens systems (Siemens Healthiness, Erlangen, Germany); Sysmex system (Sysmex, Milton Keynes, UK).

**Table 2:** Performance results of the top 5 models, * marks the best performing model.

Dataset Assessed	Features Included	No. of Features (n)	Accuracy (95% CI)	Cohen’s Kappa (%)	Sensitivity (%)	Specificity (%)	PPV (%)	NPV (%)	Ensemble RF Mean Resample Accuracy [Min, Max] (%)
Validation dataset	LM (-rbc, -tp, -glob) + CS + effusion info + PlasC + MstC + MesC	25	96.32 (91.63–98.8)	92.62	98.39	94.59	93.85	98.59	
	LM (-rbc, -tp, -glob) + CS + effusion site and type	21	96.32 (91.63–98.8)	92.62	98.39	94.59	93.85	98.59	
	LM (-rbc, -tp, -glob) + CS + effusion site, type and bicavitary	22	96.32 (91.63–98.8)	92.62	98.39	94.59	93.85	98.59	
	LM (-rbc, -tp, -glob) + CS + effusion site + bicavitary	23	96.32 (91.63–98.8)	92.62	98.39	94.59	93.85	98.59	
*	LM (-rbc, -tp, -glob) + CS + effusion site	20	96.32 (91.63–98.8)	92.62	98.39	94.59	93.85	98.59	
Testing dataset	LM (-rbc, -tp, -glob) + CS, + effusion info + PlasC + MstC + MesC	25	96.51 (92.56–98.71)	93.02	98.85	94.12	94.51	98.77	94.02 [89.8, 96.52]
	LM (-rbc, -tp, -glob) + CS + effusion site and type	21	96.51 (92.56–98.71)	93.02	98.85	94.12	94.51	98.77	94.64 [91.2, 96.26]
	LM (-rbc, -tp, -glob) + CS + effusion site, type and bicavitary	22	96.51 (92.56–98.71)	93.02	98.85	94.12	94.51	98.77	94.69 [90.4, 96.78]
	LM (-rbc, -tp, -glob) + CS + effusion site + bicavitary	23	96.51 (92.56–98.71)	93.02	98.85	94.12	94.51	98.77	94.81 [92.51, 96.52]
*	LM (-rbc, -tp, -glob) + CS + effusion site	20	96.51 (92.56–98.71)	93.02	98.85	94.12	94.51	98.77	94.82 [92.53, 96.28]

LM = laboratory markers; rbc = Fluid RBC; tp = Fluid total protein; glob = Fluid globulin; CS = All clinical signs; Effusion info = site, type and bicavitary; PlasC = Plasma cell count; MstC = Mast cell count; MesC = Mesothelial cell count; PPV = positive predictive value; NPV = pegative predictive value.

**Table 3:** Confusion matrix evaluating the performance of the best performing ensemble on the validation dataset.

		Reference		
		FIP	Non-FIP	
**Prediction**	FIP	61	4	Sensitivity 98.39%
	Non-FIP	1	70	Specificity 94.59%
		PPV 93.85%	NPV 98.59%	Accuracy 96.32% (91.63–98.8%)

PPV = positive predictive value; NPV = negative predictive value.

**Table 4:** Confusion matrix evaluating the performance of the best performing ensemble on the testing dataset.

		Reference		
		FIP	Non-FIP	
**Prediction**	FIP	86	5	Sensitivity 98.85%
	Non-FIP	1	80	Specificity 94.12%
		PPV 94.51%	NPV 98.77%	Accuracy 96.51% (92.56–98.71%)

PPV = positive predictive value; NPV = negative predictive value.

## Data Availability

The clinically sensitive data used in this study can be made available to academic institutions and other interested parties upon request. To obtain data a formal request should be made to the corresponding author. Code used in the study is available on GitHub at the following link: https://github.com/ddunbar84/Dunbar_FIP_Effusive_ML (uploaded on 15 October 2025). The script may also be made available by request.

## Supplementary Materials

The following supporting information can be downloaded at https://www.mdpi.com/article/10.3390/bioengineering13020127/s1.
