# Supplementary material for: Applying Supervised Machine Learning to Effusion Analysis for the Diagnosis of Feline Infectious Peritonitis
Source: Bioengineering (Basel). 2026 Jan 23;13(2):127. doi: 10.3390/bioengineering13020127 (PMC12937978; doi:10.3390/bioengineering13020127)
Supplement: Supplementary file 1 [file bioengineering-13-00127-s001.zip › bioengineering-3993816-supplementary.pdf]

bioengineering

Supplementary Materials

Supplementary Material S1 - Hyperparameter grid settings for modelling.

	Algorithm name	Algorithm type	Tuning hyperparameter	Hyperparameter setting
Base Learners	Logistic regression	Regression		
	Naïve Bayes	Probabilistic	laplace<br>usekernel<br>adjust	0<br>True, False<br>0, 0.25, 0.5, 0.75, 1
	Support Vector Machine	Discriminative classifier	Cost	0, 0.01, 0.05, 0.1, 0.25, 0.5, 0.75, 1, 1.25, 1.5, 1.75, 2,5
	Random Forest	Tree-based	mtry	1:11
	Xtreme Gradient Boosting	Ensemble Tree-based	nrounds<br>eta<br>max_depth<br>gamma<br>colsample_bytree<br>min_child_weight<br>subsample	200, 400, 600, 800, 1000<br>0.05<br>3,4,5<br>0<br>1<br>1<br>0.5,0.75
Ensemble	Random Forest	Tree-based	mtry	40:50

 https://doi.org/10.3390/bioengineering13020127

Supplementary Material S2 - Packages used in R for data analysis, visualisation, modelling and evaluation.

Base R packages	Additional packages
Base	table1_1.5.1
Parallel, grid, stats, graphics, grDevices, utils,	htmlTable_2.4.0,
datasets, methods	factoextra_1.0.7
	furrr_0.2.3
	future_1.23.0
	fastDummies_1.6.3
	shapr_0.2.0
	caretEnsemble_2.0.1
	doParallel_1.0.14
	iterators_1.0.13
	foreach_1.5.1
	ggbiplot_0.55
	scales_1.4.0
	devtools_2.4.3
	usethis_2.1.5
	ggfortify_0.4.14
	ggthemes_4.2.4
	ggridges_0.5.3
	gridExtra_2.3
	ggpubr_0.4.0
	Rmisc_1.5
	plyr_1.8.6
	quanteda_3.2.0
	reticulate_1.22
	randomForest_4.6-14
	xgboost_1.5.0.2
	kernlab_0.9-29
	e1071_1.7-9
	gbm_2.1.8
	pROC_1.18.0
	stringi_1.7.6
	caret_6.0-90
	lattice_0.20-45
	magrittr_2.0.3
	dataMaid_1.4.1
	knitr_1.50
	lubridate_1.9.2
	forcats_1.0.1
	stringr_1.5.2
	dplyr_1.1.4
	purrr_1.0.1

https://doi.org/10.3390/bioengineering13020127

	readr_2.1.4<br>tidyr_1.3.0<br>tibble_3.2.1<br>ggplot2_4.0.0<br>tidyverse_2.0.0

Supplementary Material S3 - Metrics from all 39 models as measured on the validation dataset. LM = laboratory markers; rbc = Fluid RBC; tp= Fluid total protein; glob = Fluid globulin; CS = All clinical signs; Effusion info = site, type and bicavitary; PlasC = Plasma cell count; MstC = Mast cell count; MesC = Mesothelial cell count; Neut = Neutrophil count; Mac = Macrophage count; Lymph = Lymphocyte count; DCCs = all 7 differential cell counts, 4DCCs = Neutrophil, Macrophage, Lymphocyte and Eosinophil differential cells counts; PPV = Positive predictive value; NPV = Negative predictive value.

Feature List	No features	Mean Accuracy	Min Accuracy	Max Accuracy	Kappa	Sensitivity	Specificity	PPV	NPV
LM + DCCs	19	95.59	90.64	98.36	91.15	98.39	93.24	92.42	98.57
LM + 4DCCs	16	95.59	90.64	98.36	91.15	98.39	93.24	92.42	98.57
LM + effusion type and site	14	95.59	90.64	98.36	91.15	98.39	93.24	92.42	98.57
LM + CS -rbc, -tp, -glob, + effusion site, bicavitary + DCCs	28	95.59	90.64	98.36	91.15	98.39	93.24	92.42	98.57
LM + CS -rbc + effusion info + DCCs	29	95.59	90.64	98.36	91.15	98.39	93.24	92.42	98.57
LM + effusion info + DCCs	22	95.59	90.64	98.36	91.15	98.39	93.24	92.42	98.57
LM + bicavitary + eff site	14	95.59	90.64	98.36	91.15	98.39	93.24	92.42	98.57
LM + bicavitary	13	95.59	90.64	98.36	91.15	98.39	93.24	92.42	98.57
LM + effusion site	13	95.59	90.64	98.36	91.15	98.39	93.24	92.42	98.57
LM only	12	95.59	90.64	98.36	91.15	98.39	93.24	92.42	98.57
LM + effusion type	13	95.59	90.64	98.36	91.15	98.39	93.24	92.42	98.57
LM + CS -rbc, -tp, -glob, + effusion info + DCCs	29	95.59	90.64	98.36	91.15	98.39	93.24	92.42	98.57
LM + CS -rbc, -tp, -glob, + effusion type, site + DCCs	28	95.59	90.64	98.36	91.15	98.39	93.24	92.42	98.57
LM + CS -rbc, -tp, -glob, + effusion site and type + DCCs	28	95.59	90.64	98.36	91.15	98.39	93.24	92.42	98.57

https://doi.org/10.3390/bioengineering13020127

LM + CS -rbc, -tp, -glob, + effusion info + 4DCCs	26	95.59	90.64	98.36	91.15	98.39	93.24	92.42	98.57
LM + bicavitary and type	14	95.59	90.64	98.36	91.15	98.39	93.24	92.42	98.57
LM + CS + effusion info + DCCs (all features)	32	96.32	91.63	98.8	92.62	98.39	94.59	93.85	98.59
LM + effusion info + 4DCCs	19	96.32	91.63	98.8	92.62	98.39	94.59	93.85	98.59
LM + CS -rbc, -tp, -glob, + effusion info + Neut, Mac, Lymph	25	96.32	91.63	98.8	92.62	98.39	94.59	93.85	98.59
LM + CS -rbc, -tp, -glob, + effusion info + Neut, Mac	24	96.32	91.63	98.8	92.62	98.39	94.59	93.85	98.59
LM + CS -rbc -tp	20	96.32	91.63	98.8	92.62	98.39	94.59	93.85	98.59
LM + CS -rbc -tp -glob	19	96.32	91.63	98.8	92.62	98.39	94.59	93.85	98.59
LM + CS -rbc, -tp, -glob, + effusion type, bicavitary + DCCs	30	96.32	91.63	98.8	92.62	98.39	94.59	93.85	98.59
LM + CS -rbc, -tp, -glob, + effusion site + DCCs	27	96.32	91.63	98.8	92.62	98.39	94.59	93.85	98.59
LM + CS -rbc, -tp, -glob, + effusion site and type + 4DCCs	25	96.32	91.63	98.8	92.62	98.39	94.59	93.85	98.59
LM + CS	22	96.32	91.63	98.8	92.62	98.39	94.59	93.85	98.59
LM + CS -rbc, -tp, -glob, + effusion info + Neut	21	96.32	91.63	98.8	92.62	98.39	94.59	93.85	98.59
LM + CS -rbc, -tp, -glob, + effusion info + PlasC, MstC, MesC	25	96.32	91.63	98.8	92.62	98.39	94.59	93.85	98.59
LM + CS -rbc, -tp, -glob, + effusion site and type	21	96.32	91.63	98.8	92.62	98.39	94.59	93.85	98.59
LM + CS -rbc, -tp, -glob, + effusion info	22	96.32	91.63	98.8	92.62	98.39	94.59	93.85	98.59
LM + CS -rbc, -tp, -glob, + effusion site + bicavitary	23	96.32	91.63	98.8	92.62	98.39	94.59	93.85	98.59
LM + CS -rbc, -tp, -glob, + effusion site	20	96.32	91.63	98.8	92.62	98.39	94.59	93.85	98.59
LM + CS -rbc, -tp, + effusion info + DCCs	29	97.06	92.64	99.19	94.09	98.39	95.95	95.31	98.61
LM + CS -tp, + effusion info + DCCs	31	97.06	92.64	99.19	94.09	98.39	95.95	95.31	98.61
LM + CS -rbc, -tp, -glob, + effusion type + DCCs	27	97.06	92.64	99.19	94.09	98.39	95.95	95.31	98.61

https://doi.org/10.3390/bioengineering13020127

LM + CS -rbc	21	97.06	92.64	99.19	94.09	98.39	95.95	95.31	98.61
LM + CS -rbc, -tp, -glob, + effusion site and type + Neut	22	97.06	92.64	99.19	94.09	98.39	95.95	95.31	98.61
LM + CS -rbc, -tp, -glob, + effusion type + bicavitary	21	97.06	92.64	99.19	94.09	98.39	95.95	95.31	98.61
LM + CS -rbc, -tp, -glob, + effusion type	20	97.06	92.64	99.19	94.09	98.39	95.95	95.31	98.61

Supplementary Material S4 - Metrics from all 39 models as measured on the testing dataset. LM = laboratory markers; rbc = Fluid RBC; tp= Fluid total protein; glob = Fluid globulin; CS = All clinical signs; Effusion info = site, type and bicavitary; PlasC = Plasma cell count; MstC = Mast cell count; MesC = Mesothelial cell count; Neut = Neutrophil count; Mac = Macrophage count; Lymph = Lymphocyte count; DCCs = all 7 differential cell counts, 4DCCs = Neutrophil, Macrophage, Lymphocyte and Eosinophil differential cells counts; PPV = Positive predictive value; NPV = Negative predictive value.

Feature List	No Features	Mean Accuracy	Min Accuracy	Max Accuracy	Kappa	Sensitivity	Specificity	PPV	NPV
LM + DCCs	19	94.77	90.3	97.58	89.53	97.7	91.76	92.39	97.5
LM + 4DCCs	16	94.77	90.3	97.58	89.53	97.7	91.76	92.39	97.5
LM + effusion type and site	14	94.77	90.3	97.58	89.53	97.7	91.76	92.39	97.5
LM + CS -rbc, -tp, -glob, + effusion site, bicavitary + DCCs	28	95.35	91.04	97.97	90.69	98.85	91.76	92.47	98.73
LM + CS -rbc + effusion info + DCCs	29	95.35	91.04	97.97	90.69	98.85	91.76	92.47	98.73
LM + effusion info + DCCs	22	95.35	91.04	97.97	90.69	97.7	92.94	93.41	97.53
LM + bicavitary + eff site	14	95.35	91.04	97.97	90.69	97.7	92.94	93.41	97.53
LM + bicavitary	13	95.35	91.04	97.97	90.69	97.7	92.94	93.41	97.53
LM + effusion site	13	95.35	91.04	97.97	90.69	97.7	92.94	93.41	97.53
LM only	12	95.35	91.04	97.97	90.69	97.7	92.94	93.41	97.53
LM + effusion type	13	95.35	91.04	97.97	90.69	97.7	92.94	93.41	97.53
LM + CS -rbc, -tp, -glob, + effusion info + DCCs	29	95.93	91.79	98.35	91.85	100	91.76	92.55	100
LM + CS -rbc, -tp, -glob, + effusion type, site + DCCs	28	95.93	91.79	98.35	91.85	100	91.76	92.55	100
LM + CS -rbc, -tp, -glob, + effusion site and type + DCCs	28	95.93	91.79	98.35	91.85	100	91.76	92.55	100
LM + CS -rbc, -tp, -glob, + effusion info + 4DCCs	26	95.93	91.79	98.35	91.85	100	91.76	92.55	100
LM + bicavitary and type	14	95.93	91.79	98.35	91.85	98.85	92.94	93.48	98.75

https://doi.org/10.3390/bioengineering13020127

LM + CS + effusion info + DCCs (all features)	32	95.35	91.04	97.97	90.69	97.7	92.94	93.41	97.53
LM + effusion info + 4DCCs	19	95.35	91.04	97.97	90.69	97.7	92.94	93.41	97.53
LM + CS -rbc, -tp, -glob, + effusion info + Neut, Mac, Lymph	25	95.35	91.04	97.97	90.69	98.85	91.76	92.47	98.73
LM + CS -rbc, -tp, -glob, + effusion info + Neut, Mac	24	95.35	91.04	97.97	90.69	98.85	91.76	92.47	98.73
LM + CS -rbc -tp	20	95.35	91.04	97.97	90.69	97.7	92.94	93.41	97.53
LM + CS -rbc -tp -glob	19	95.35	91.04	97.97	90.69	97.7	92.94	93.41	97.53
LM + CS -rbc, -tp, -glob, + effusion type, bicavitary + DCCs	30	95.93	91.79	98.35	91.85	98.85	92.94	93.48	98.75
LM + CS -rbc, -tp, -glob, + effusion site + DCCs	27	95.93	91.79	98.35	91.85	98.85	92.94	93.48	98.75
LM + CS -rbc, -tp, -glob, + effusion site and type + 4DCCs	25	95.93	91.79	98.35	91.85	100	91.76	92.55	100
LM + CS	22	95.93	91.79	98.35	91.86	97.7	94.12	94.44	97.56
LM + CS -rbc, -tp, -glob, + effusion info + Neut	21	95.93	91.79	98.35	91.85	98.85	92.94	93.48	98.75
LM + CS -rbc, -tp, -glob, + effusion info + PlasC, MstC, MesC	25	96.51	92.56	98.71	93.02	98.85	94.12	94.51	98.77
LM + CS -rbc, -tp, -glob, + effusion site and type	21	96.51	92.56	98.71	93.02	98.85	94.12	94.51	98.77
LM + CS -rbc, -tp, -glob, + effusion info	22	96.51	92.56	98.71	93.02	98.85	94.12	94.51	98.77
LM + CS -rbc, -tp, -glob, + effusion site + bicavitary	23	96.51	92.56	98.71	93.02	98.85	94.12	94.51	98.77
LM + CS -rbc, -tp, -glob, + effusion site	20	96.51	92.56	98.71	93.02	98.85	94.12	94.51	98.77
LM + CS -rbc, -tp, + effusion info + DCCs	29	95.35	91.04	97.97	90.69	98.85	91.76	92.47	98.73
LM + CS -tp, + effusion info + DCCs	31	95.93	91.79	98.35	91.85	98.85	92.94	93.48	98.75
LM + CS -rbc, -tp, -glob, + effusion type + DCCs	27	95.93	91.79	98.35	91.85	98.85	92.94	93.48	98.75
LM + CS -rbc	21	95.93	91.79	98.35	91.86	97.7	94.12	94.44	97.56
LM + CS -rbc, -tp, -glob, + effusion site and type + Neut	22	95.93	91.79	98.35	91.85	98.85	92.94	93.48	98.75

LM + CS -rbc, -tp, -glob, + effusion type + bicavitary	21	95.93	91.79	98.35	91.85	98.85	92.94	93.48	98.75
LM + CS -rbc, -tp, -glob, + effusion type	20	95.93	91.79	98.35	91.85	98.85	92.94	93.48	98.75

Supplementary Material S5 - Accuracy measures from the final ensemble randomForest model, for all 39 iterative models. LM = laboratory markers; rbc = Fluid RBC; tp= Fluid total protein; glob = Fluid globulin; CS = All clinical signs; Effusion info = site, type and bicavitary; PlasC = Plasma cell count; MstC = Mast cell count; MesC = Cesothelial cell count; Neut = Neutrophil count; Mac = Macrophage count; Lymph = Lymphocyte count; DCCs = all 7 differential cell counts, 4DCCs = Neutrophil, Macrophage, Lymphocyte and Eosinophil differential cells counts; PPV = Positive predictive value; NPV = Negative predictive value.

Feature List	No Features	Mean ensemble Accuracy	Min ensemble Accuracy	Max ensemble Accuracy
LM + DCCs	19	93.17	83.93	96.25
LM + 4DCCs	16	93.85	87.72	96.51
LM + effusion type and site	14	94.87	92.8	96.51
LM + CS -rbc, -tp, -glob, + effusion site, bicavitary + DCCs	28	92.85	82.35	96.79
LM + CS -rbc + effusion info + DCCs	29	92.87	84.74	96.28
LM + effusion info + DCCs	22	92.92	82.62	96.53
LM + bicavitary + eff site	14	94.72	93.29	96.25
LM + bicavitary	13	94.8	92.8	96.25
LM + effusion site	13	94.86	93.56	96.23
LM only	12	94.92	93.58	96.51
LM + effusion type	13	95	93.58	96.26
LM + CS -rbc, -tp, -glob, + effusion info + DCCs	29	92.76	82.62	96.79
LM + CS -rbc, -tp, -glob, + effusion type, site + DCCs	28	93.04	83.93	96.79
LM + CS -rbc, -tp, -glob, + effusion site and type + DCCs	28	93.04	83.93	96.79
LM + CS -rbc, -tp, -glob, + effusion info + 4DCCs	26	93.7	88.24	96.79
LM + bicavitary and type	14	94.96	93.31	96.79
LM + CS + effusion info + DCCs (all features)	32	92.7	84.22	96.13
LM + effusion info + 4DCCs	19	93.67	87.69	96.26
LM + CS -rbc, -tp, -glob, + effusion info + Neut, Mac, Lymph	25	94.08	90.11	97.04
LM + CS -rbc, -tp, -glob, + effusion info + Neut, Mac	24	94.37	90.65	96.78
LM + CS -rbc -tp	20	94.69	92.81	96.53
LM + CS -rbc -tp -glob	19	94.92	92.82	96.51
LM + CS -rbc, -tp, -glob, + effusion type, bicavitary + DCCs	30	92.82	82.89	96.79
LM + CS -rbc, -tp, -glob, + effusion site + DCCs	27	93.1	84.2	96.79
LM + CS -rbc, -tp, -glob, + effusion site and type + 4DCCs	25	93.9	88.77	96.79
LM + CS	22	94.24	91.45	96.25
LM + CS -rbc, -tp, -glob, + effusion info + Neut	21	94.46	91.44	96.51
LM + CS -rbc, -tp, -glob, + effusion info + PlasC, MstC, MesC	25	94.02	89.82	96.52
LM + CS -rbc, -tp, -glob, + effusion site and type	21	94.64	91.2	96.26

https://doi.org/10.3390/bioengineering13020127

LM + CS -rbc, -tp, -glob, + effusion info	22	94.69	90.4	96.78
LM + CS -rbc, -tp, -glob, + effusion site + bicavitary	23	94.81	92.51	96.52
LM + CS -rbc, -tp, -glob, + effusion site	20	94.82	92.53	96.28
LM + CS -rbc, -tp, + effusion info + DCCs	29	92.88	84.47	96.52
LM + CS -tp, + effusion info + DCCs	31	92.68	83.42	96.53
LM + CS -rbc, -tp, -glob, + effusion type + DCCs	27	93.1	84.2	96.79
LM + CS -rbc	21	94.34	91.68	96.26
LM + CS -rbc, -tp, -glob, + effusion site and type + Neut	22	94.47	91.2	96.79
LM + CS -rbc, -tp, -glob, + effusion type + bicavitary	21	94.75	91.46	96.27
LM + CS -rbc, -tp, -glob, + effusion type	20	94.8	91.99	96.27

https://doi.org/10.3390/bioengineering13020127
